# Liver Transplantation in Patients with Hepatocellular Carcinoma beyond the Milan Criteria: A Comprehensive Review

**DOI:** 10.3390/jcm10173932

**Published:** 2021-08-31

**Authors:** Pierluigi Toniutto, Elisa Fumolo, Ezio Fornasiere, Davide Bitetto

**Affiliations:** Hepatology and Liver Transplantation Unit, Azienda Sanitaria Universitaria Integrata, 33100 Udine, Italy; elisa.fumolo@asufc.sanita.fvg.it (E.F.); ezio.fornasiere@asufc.sanita.fvg.it (E.F.); davide.bitetto@asufc.sanita.fvg.it (D.B.)

**Keywords:** hepatocellular carcinoma, liver transplantation, Milan criteria, alpha-fetoprotein

## Abstract

The Milan criteria (MC) were developed more than 20 years ago and are still considered the benchmark for liver transplantation (LT) in patients with hepatocellular carcinoma (HCC). However, the strict application of MC might exclude some patients who may receive a clinical benefit of LT. Several expanded criteria have been proposed. Some of these consider pretransplant morphological and biological variables of the tumor, others consider post-LT variables such as the histology of the tumor, and others combine pre- and post-LT variables. More recently, the HCC response to locoregional treatments before transplantation emerged as a surrogate marker of the biological aggressiveness of the tumor to be used as a better selection criterion for LT in patients beyond the MC at presentation. This essential review aims to present the current data on the pretransplant selection criteria for LT in patients with HCC exceeding the MC at presentation based on morphological and histological characteristics of the tumor and to critically discuss those that have been validated in clinical practice. Moreover, the role of HCC biological markers and the tumor response to downstaging procedures as new tools for selecting patients with a tumor burden outside of the MC for LT is evaluated.

## 1. Introduction

Hepatocellular carcinoma (HCC) is the fifth most common cancer and the third most common cause of cancer-related mortality [1]. The incidence of HCC has progressively increased during recent decades due to the increased number of patients with liver cirrhosis caused by chronic hepatitis C (HCV), hepatitis B (HBV), and alcohol abuse, as well as patients with metabolic syndrome-induced liver disease [2]. Fewer than half of HCC cases are diagnosed at an early stage, which allows them to receive curative treatments such as surgical resection, locoregional ablative therapy, and liver transplantation (LT) [3]. LT is considered the best treatment option for HCC because removal of the native liver simultaneously eliminates the tumor and the underlying liver disease [4].

In the second half of the 1960s, LT programs were developed in humans with the aim of offering radical treatment of unresectable liver tumors [5]. However, the initial enthusiasm for these experiences quickly dwindled, as it appeared evident that post-transplant survival was unsatisfactory due to the unsustainable tumor recurrence rates in the transplanted liver, which predicted poor survival [6]. These unsatisfactory results stemmed from a lack of precise selection criteria for patients to undergo transplantation, which were based on the tumor type and on the intrahepatic burden of the neoplastic disease [7]. Thus, the dramatic change that allowed improvement of the post-transplant survival in patients with HCC was the introduction of more accurate selection criteria. In 1996, Mazzaferro et al. published for the first time the Milan criteria (MC), which are still largely used as the reference benchmark to select patients with HCC for LT in many countries. The MC were based on HCC morphological characteristics evaluable before LT (up to three HCC nodules the largest < 3 cm in diameter or a single HCC nodule up to 5 cm in diameter), without macrovascular invasion or extrahepatic spread of the tumor [8]. Patients fulfilling MC experienced a 4-year survival rate of 75%. Several studies confirmed these results and demonstrated that the overall post-transplant survival of HCC patients transplanted within MC was not unlike that of patients transplanted for decompensated liver cirrhosis without HCC [9]. However, even in patients transplanted within MC, HCC recurrence was described in 10–16% of cases and was the main cause of death [10,11]. These data showed that despite the careful selection of HCC patients for transplantation, based on tumor morphological criteria, the risk of HCC recurrence remained consistent. This may be explained by the dissemination of circulating cancer cells and micrometastases before or during transplant operation [12]. Thus, the key issue in the success of LT for HCC is to select candidates that present as the least likely to experience tumor recurrence after transplantation and who maintain a comparable post-LT survival expectancy to that of non-HCC recipients [13].

**Figure 1:**
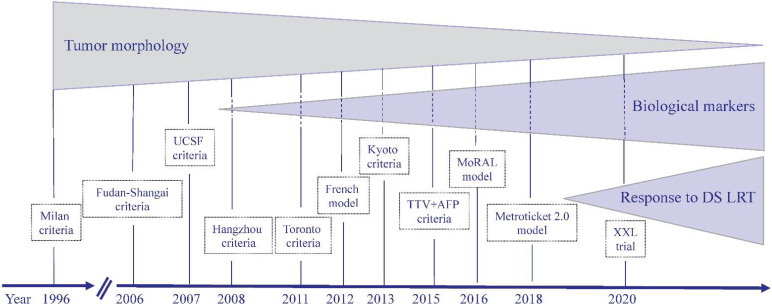
Evolving concepts in the selection of patients with HCC for liver transplantation. After the discovery of the Milan criteria in 1996, the subsequently expanded criteria for liver transplantation in patients with HCC were mainly based on the morphological characteristics of the tumor. Starting in 2008, with the Hangzhou criteria, the addition of biological markers to tumor morphologic criteria allowed expansion of the original Milan criteria, maintaining a good clinical outcome of liver transplanted patients with HCC. More recently, the new way to select patients to be transplanted for HCC who are beyond the Milan criteria at presentation has been to qualify those who can be traced back to the Milan criteria after successful downstaging after locoregional and/or systemic treatments. UCSF: University California, San Francisco; TTV: total tumor volume; AFP: alpha-fetoprotein; DS: downstaging; LRT: locoregional treatment.

Although the MC are still largely applied to candidate LT patients with HCC, a growing number of studies have shown that acceptable post-LT may be obtained in patients exceeding the MC at baseline [14,15,16,17]. This evidence is of paramount importance, suggesting that the strict application of the MC in all LT candidates could take away the possibility of transplantation in some patients who instead would have an important clinical benefit of LT [18]. The reasons why the MC did not allow accurate prediction of the outcome after LT in all patients stem from the fact that they were based exclusively on tumor morphological characteristics, such as the size and number of nodules. To overcome this limitation, several selection criteria that sought to expand the MC were proposed [19]. Some of these criteria were constructed using morphological and biological variables of the tumor obtainable pretransplant (Table 1 and Table 2), others on variables obtainable only after transplantation (for example, the full histology of the tumor), and others by combining variables obtainable in both the pre- and post-transplantation periods. More recently, it has been proposed that the response of HCC to locoregional treatment (LRT) before transplantation may represent a surrogate marker of biological aggressiveness of the tumor to be used to improve patient selection for transplantation and overcome the limitations of criteria based exclusively on tumor morphology. This concept shifts the current paradigm to select for LT-only patients fulfilling the MC at baseline and offers the possibility of considering all patients with an HCC outside the MC at baseline that may be downstaged with locoregional treatments to within the MC as potentially suitable for LT (Figure 1).

It should be considered that the expansion of the transplant criteria for HCC will progressively increase the number of potential candidates on the waiting list. This imposes three main questions when contemplating expansion of the MC for LT in patients with HCC: (a) What upper limit of the tumor burden beyond the MC could be accepted for LT? (b) What could be the minimal acceptable overall survival after LT? (c) How could markers of tumor biology, in addition to the tumor burden, be incorporated to better select patients for LT?

This essential review aims to present the current data on the pretransplant selection criteria for LT in patients with HCC exceeding the MC at presentation based on morphological and histological characteristics of the tumor and to critically discuss those that have been validated in clinical practice. Moreover, the role of HCC biological markers and the tumor response to downstaging procedures as new tools for selecting patients for LT with a baseline tumor burden outside of the MC will be evaluated.

## 2. Selection Criteria for LT Based on HCC Morphological Characteristics

A common theme in the morphological criteria adopted to select patients with HCC was to exclude those patients presenting HCC with macrovascular invasion and/or extrahepatic spread. The main reason why morphological pre-LT criteria improved the accuracy in the selection of patients with HCC for LT derives from the demonstration that both the number and the size of HCC nodules can be considered surrogate markers of histologic microvascular invasion (MVI) and/or poor tumor differentiation [28,29], which are the main determinants of HCC recurrence and death after LT [30]. In addition to MC, two pretransplant expansive morphological criteria were adopted and validated in cohorts of patients of various geographical origins. The Shanghai Fudan criteria were originally developed in patients with chronic HBV infection. Patients transplanted with a single HCC nodule ≤ 9 cm in diameter or with up to three lesions (the largest ≤ 5 cm) but with a maximum tumor diameter of ≤9 cm experienced 1- and 3-year post-LT survival and HCC recurrence-free survival comparable with those transplanted within the MC [20]. These criteria were subsequently validated in more than 1000 patients enrolled in liver transplant centers in Shanghai [31]. In the United States, a further criterion aimed at expanding the original MC was developed at the University of California, San Francisco (UCSF) [14]. The UCSF criteria were developed after the observation that patients transplanted with a single nodule of HCC ≤ 6.5 cm or with up to 2–3 lesions ≤ 4.5 cm each, maintaining the total tumor diameter ≤ 8 cm, experienced a 5-year post-LT recurrence-free survival of 80.7%, which was not significantly different than that obtained in patients transplanted within the MC.

Both new criteria increased, albeit slightly, the size and/or the number of HCC nodules to select patients for LT compared to what was originally established by the MC. The certainty of always being able to carry out an accurate and reproducible measurement of the size and number of nodules to capture millimeter differences with respect to the MC probably represents the greatest limitation of the new criteria based exclusively on the morphological characteristics of HCC. There is great heterogeneity and different accuracies of liver imaging techniques applied to detect liver nodules and to properly characterize them, such as contrast-enhanced computed tomography (CT) or magnetic resonance imaging (MRI) [19]. Several reports have indicated that as many as 20–25% of patients undergoing LT for HCC have been inaccurately staged when only imaging techniques were used [32,33]. To overcome this important drawback, the American College of Radiology created the Liver Imaging Reporting and Data System (LI-RADS), aimed at standardizing the process of acquisition, interpretation, reporting, and data collection for liver imaging [34]. LI-RADS stratified the characteristics of liver lesions into five categories starting from LR-1 (definitely benign) to LR-5 (definitely HCC). This system is adopted in many countries to allow a standardized differential diagnosis of liver nodules in patients at high risk of HCC. Despite this improvement in the radiological categorization of liver nodules, a recent meta-analysis showed that LI-RADS was only 67% sensitive and 92% specific in diagnosing HCC [35].

## 3. Selection Criteria for LT Based on HCC Histological Characteristics

In patients transplanted beyond MC, a greater incidence of MVI, which is associated with higher post-LT tumor recurrence and death, has been demonstrated [36]. When the explanted livers of patients within and outside the MC were compared, MVI was present in 11% of the former group and in 42% of the latter group [37]. A potential tool to detect the presence of MVI of HCC before LT is to perform liver biopsy of the nodules. Liver biopsy of confirmed HCC in the explanted liver was performed before LT in a series of 155 patients [33]. MVI was significantly more frequent in patients with larger nodules or with multinodular HCC; furthermore, 68% of patients who experienced HCC recurrence after LT were positive for MVI. In addition to the detection of MVI, another potential tool employing liver biopsy of HCC is to evaluate tumor histological grading. It has been demonstrated that well-differentiated HCCs may be successfully transplanted, assuring a recipient 5-year post-LT survival of 75% despite approximately 30% of these patients being beyond MC at the explant [38]. Although the clinical usefulness of pre-LT histologic assessment of tumor grading and MVI may be important, routine biopsy of HCC is not often feasible, mainly due to the presence of multiple nodules or the risk of promoting seeding of cancer cells [39].

The current challenging question is whether the presence of MVI may be predicted by means of noninvasive methods. To answer this question, several authors propose the use of imaging techniques such 18F-FDG PET/CT or gadoxetic acid enhanced dynamic MRI as noninvasive methods to predict the presence of MVI [40,41]. The rationale for using 18F-FDG PET/CT in detecting MVI derives from the observation that the HCC growth rate and the activity of glycolytic enzymes are related [42]. This explains why poorly differentiated HCC shows low glucose-6 phosphatase activity and high 18F-FDG uptake [43]. It has been proposed that good cutoff values for the SUVmax of HCC (SUVmax T) and SUVmax of the normal liver (SUVmax L) in predicting the presence of MVI may be 3.80 and 1.49, respectively [44]. The SUVmax T/SUVmean L ratio ≥ 1.2 was demonstrated to be significantly associated with the presence of MVI [45]. Compared with 18F-FDG PET/CT alone, the addition of gadoxetic acid enhanced dynamic MRI is a promising technology that may further improve the sensitivity and specificity of MVI detection. This was confirmed by applying the combination of MRI and PET/CT, as the sensitivity and specificity in predicting the presence of MVI were 78.6% and 80%, respectively [40]. These observations have been confirmed in clinical studies conducted in Asia in living donor liver transplantation. In Japan, 182 living donor liver transplanted patients with HCC were studied by means of 18F-FDG PET/CT and the serum levels of alpha-fetoprotein (AFP) before LT. In recipients transplanted beyond MC who presented negative 18F-FDG PET/CT and AFP serum levels < 115 ng/mL, the 5-year HCC recurrence rate was comparable to those transplanted within MC [46]. Very similar results were obtained in a Korean study also conducted in patients transplanted outside the Milan and UCSF criteria using living donors. The authors demonstrated that these two groups of patients, if they presented a negative 18F-FDG PET/CT, experienced 5-year post-LT HCC-free survival rates of 73.3% and 72.8%, respectively [47]. Although these results are encouraging, it is necessary to confirm them in populations of different ethnic groups, where the causes of LT are often different from those present in Asia, as well as in patients transplanted using deceased donors. At the present time, the combined use of 18F-FDG PET/CT and gadoxetic acid enhanced MRI could be applied in addition to the morphological and biological characteristics of HCC to stratify the risk of MVI.

## 4. Selection Criteria Based on Serum Biological Marker Measurements

The measurement of serum markers referring to biological tumor characteristics and/or to host immune system reactivity has been considered a fascinating approach to overcome the limits of morphological criteria. Three main categories of measurable biological markers are currently available: (a) serum markers related to the biological characteristics of HCC, such as AFP and des-gamma-carboxyprothrombin (DCP); (b) markers reflecting systemic host inflammation (neutrophil-to-lymphocyte ratio and platelet-to-lymphocyte ratio); and (c) molecular biomarkers that may be measured both in liver tissue and in serum (genetic mutations, enzymes, and microRNAs). In relation to the clinical purpose of this review, only serum markers will be discussed, as there is still no solid evidence justifying the use of inflammation and molecular biomarkers in clinical practice.

## 5. Selection Criteria Based on the Addition of AFP and/or DCP Serum Level Measurements to HCC Morphology

AFP is considered a marker of HCC differentiation and vascular invasion; thus, the measurement of its serum levels pre-LT has been proposed as a potential tool to identify HCC patients with a higher risk of tumor recurrence and poor post-LT survival who should be excluded from transplantation [48]. The limitations of this approach became immediately evident, since it was very difficult to apply AFP measurement in a standardized and reproducible timeframe. For example, in deceased donor liver transplantation, the date of the transplant is never predictable. Thus, the optimal time interval between transplantation and AFP measurement that would make it a predictive marker of recurrence was unclear [19]. Instead of adopting cutoff values of AFP, an interesting approach is to consider multiple AFP measurements to calculate a trend in the increase or decrease in its serum levels. Patients experiencing an increase in serum AFP > 15 ng/mL/month had a higher frequency of waitlist dropout or significantly worse post-LT survival than those with a lower (≤15 ng/mL/month) increase in AFP (54% vs. 94%) [49]. These results suggest that the variations in AFP serum levels, rather than the last AFP level available before LT, may be more accurate in predicting post-LT outcome. [50]. This concept is incorporated to increase the accuracy of the assessment of tumor downstaging before LT. For example, a rapid increase in AFP serum levels after a presumed successful downstaging, assessed by radiologic imaging, should be considered a predictor of poor post-transplant outcome.

A very attractive way to select in patients beyond MC at baseline those with a higher risk of HCC recurrence is to add AFP serum levels to the morphologic characteristics of HCC [51]. The Toronto criteria [21] were developed assuming that all patients with HCC may have acceptable post-LT survival independent of the number and/or size of the nodules if HCC was confined to the liver, well differentiated at histology, and without macrovascular invasion. These criteria identified AFP serum levels > 400 IU/mL at the time of transplant as an independent predictor of worse 5-year disease-free survival. The Toronto criteria were subsequently validated, confirming that AFP serum levels before LT were strongly associated with post-LT survival and HCC recurrence [52].

Similar results, combining the morphology and histology of HCC with AFP serum levels, have been obtained in China, where the Hangzhou criteria were developed [22]. These criteria selected HCC patients for LT in the absence of portal vein tumor invasion and with either HCC ≤ 8 cm in diameter or with HCC ≥ 8 cm in diameter but with concurrent AFP serum levels < 400 ng/mL and histological grade I or II. Patients fulfilling the Hangzhou criteria experienced 1- and 3-year survival rates very similar to those reported in patients transplanted within the MC. The combination of AFP serum levels and morphologic characteristics of the tumor also inspired European authors to expand the MC for selecting patients with HCC for LT. Toso et al. [53] evaluated a large cohort of 6000 European LT patients and demonstrated that the subgroup of recipients who presented a total tumor volume (TTV) of ≤115 cm^3^ and AFP serum levels ≤ 400 ng/mL experienced a lower risk of HCC recurrence and better survival after LT. It should be highlighted that these expanded criteria were more effective than both the Milan and UCSF criteria in selecting patients with a low risk of HCC recurrence for LT. The TTV-AFP criteria were subsequently validated in cohorts of patients in countries outside Europe and in Canada [23]. A further model that combined AFP serum levels and morphologic characteristics of HCC, known as the AFP model, was proposed by the Liver Transplantation French Group [24]. The AFP model merges AFP serum levels and the size and the number of nodules, attributing different scores for each variable. Tumor sizes of 0, 1, or 4 points were assigned if the largest tumor diameter was ≤3 cm, 3–6 cm, or >6 cm, respectively. Moreover, 0 or 2 points were assigned if the number of nodules was ≤3 or ≥4. Regarding the AFP serum levels, 0, 2, or 3 points were assigned if AFP serum levels were ≤100, 100–1000, or >1000 ng/mL, respectively. The maximum score sum of the AFP model is 9. Patients may be divided into low risk of HCC recurrence if the final score is up to 2 points and high risk of HCC recurrence if the final score is ≥3. A very innovative observation of the study was that in patients presenting AFP serum levels > 1000 ng/mL, 3 points are attributed, irrespective of the number or size of nodules; thus, they may be immediately considered at high risk of HCC recurrence. The AFP model was validated in different countries and in living donor liver transplantation, confirming its clinical utility in stratifying the recurrence risk of HCC after LT in a better way than MC [54,55,56,57]. The Metroticket 2.0 model [25], developed in Italy, was based on the measurement of the sum of the number and size of nodules and the log_10_ AFP level. Recipients with AFP levels < 200 ng/mL and with the sum of the number and size of tumors (in centimeters) not exceeding 7 presented a post-LT survival probability of 70%, which was comparable to that observed in patients transplanted within MC. To maintain this excellent clinical outcome in the presence of AFP levels of 200–400 ng/mL, the sum of the number and size of tumors should be reduced to ≤5, and if AFP levels are 400–1000 ng/mL, the sum of the number and size of tumors should be further reduced to ≤4. This model outperformed the original MC, UCSF, and AFP French models in identifying patients with excellent 5-year post-LT survival.

The results obtained by combining the morphological characteristics of the tumor and the AFP values made it possible to develop selection criteria for LT that definitively exceeded those of Milan. These models introduced many innovations to more accurately select patients with HCC for LT. First, these selection criteria made it possible to offer LT to many patients with HCC who would have been excluded by application of the MC, assuring excellent post-transplant survival. Second, the calculation of both the size and number of nodules and the AFP serum levels appears simple and available in every context, making these models applicable in different geographical contexts and with all types of patients. Third, these models may be used “dynamically”, in addition to the assessment of the HCC response to neoadjuvant treatments, to more accurately select patients who will undergo tumor downstaging before LT.

The interest in measuring DCP serum levels was derived from the observation that some HCCs expressed normal levels of AFP but increased levels of DCP. These subtypes of HCCs present a poor grade of differentiation and frequent MVI [58,59]. The combination of morphological characteristics of HCC (up to 10 nodules ≤ 5 cm in diameter) and DCP serum levels (≤400 mAU/mL) are the key elements included in the Kyoto criteria [26,60,61]. Patients beyond MC who fulfilled the Kyoto criteria at the time of LT had similar post-LT prognoses in terms of survival and HCC recurrence compared to those within MC [60]. In the context of living donor liver transplantation, the Kyushu criteria [62] that are considered suitable for LT patients with any number of HCC < 5 cm in diameter and DCP serum levels < 300 mAU/mL were developed. These criteria appeared more accurate for predicting HCC recurrence than both the Kyoto and UCSF criteria but only when living donation was considered [63,64].

Another interesting way to construct a prediction model of post-LT clinical outcome in patients with HCC beyond MC is to combine DCP and AFP serum levels. Starting from this assumption, the MoRAL model was developed in living donor liver transplant patients exceeding the MC [27]. Both AFP and DCP serum levels were significantly associated with the time elapsed from transplantation to HCC recurrence. In the group of recipients exceeding the MC, a MoRAL score ≤ 314.8 was predictive to select patients with significantly longer (66.3%) 5-year recurrence-free and overall (82.1%) survival. In contrast, the group of recipients fulfilling the MC but with a MoRAL score > 314.8 showed a higher risk of HCC recurrence and lower post-LT survival than patients beyond MC with a low MoRAL score. A retrospective study evaluating the combination of DCP and AFP serum levels in predicting clinical outcome in liver transplant patients outside the MC was also conducted in the United States [65]. In this study, AFP and DCP serum levels ≥ 250 ng/mL and ≥7.5 ng/mL, respectively, were predictive of more frequent HCC recurrence. When AFP and DCP were combined with MC, the hazard ratio of HCC recurrence risk increased from 2.6 for patients beyond MC to 8.6 when AFP serum levels were ≥250 ng/mL and to 7.2 when DCP serum levels were ≥7.5 ng/mL. The encouraging results deriving from the use of DCP as post-transplant survival as well as post-transplant HCC recurrence predictor should be analyzed with caution. It should be emphasized that more than 90% of the published papers related to DCP have been produced in Asian countries and refer to living donor liver transplanted patients for liver diseases mainly related to HBV infection [66]. Thus, solid data on the role of DCP in conditioning the clinical outcome of patients with HCC transplanted with deceased donors and with liver diseases due to nonviral etiologies are still lacking. Considering these limitations, among all biological markers that have been studied, AFP remains the only one that has proven useful in predicting the clinical outcome in patients transplanted for HCC. The prognostic models of post-transplant survival that incorporate pre-LT AFP serum levels and HCC morphological characteristics remain the most widely used to accurately select patients beyond MC for LT. In addition, the evaluation of AFP serum level variations induced by locoregional therapies and HCC downstaging modalities is becoming the most promising strategy to select patients to be transplanted with HCC beyond the MC with even greater accuracy.

## 6. Selection Criteria Based on the Response of HCC to Bridging and Downstaging Treatments

A very innovative approach to select patients for LT presenting at baseline beyond the MC is to evaluate the characteristics of tumor response after LRT and consider it a surrogate marker of biological HCC aggressiveness and of the risk of recurrence [19]. When LRT is used to control tumor growth with the aim of reducing the risk of waiting list dropout, it may be considered a “bridge” treatment to LT. The efficacy of LRT in reducing waiting list dropout has been demonstrated if the waiting time for LT is at least 6 months [67]. Patients whose tumor progression occurs despite LRT within 6 months have a worse post-LT outcome than those who achieved treatment response or the stability of HCC following LRT [49,68,69]. These observations suggest that the response to LRT might be influenced not only by the treatment modality but also, more importantly, by the biological behavior of the tumor.

The term “downstaging” is used by attributing the possibility of LRT decreasing the baseline tumor burden until it meets the criteria for LT (ideally within MC) and to assure acceptable post-LT outcomes [36]. This concept was derived from some studies suggesting that post-LT outcomes in patients successfully downstaged to the MC were comparable with those observed in transplanting patients with MC at presentation [70,71]. The potential explanation of these findings is that a close correlation exists between successful downstaging and better explant histologic characteristics of the tumor [70,71,72].

As indicated by the Barcelona Clinic Liver Cancer (BCLC) staging system [73], among the LRT modalities that may be employed to perform downstaging of HCC, transarterial chemoembolization (TACE) is the most commonly utilized. Transarterial radioembolization (TARE) and ablative techniques may be often proposed [74].

It should be emphasized that there are important potential safety concerns of TACE and TARE in generating hepatic decompensation. In accordance with the guidelines for TACE [3], it has been strongly suggested that only patients with preserved liver function (Child–Pugh score A/B and bilirubin ≤ 3 mg/dL) should be considered for downstaging procedures [75]. Although TACE is the recommended first-line treatment for downstaging objectives in most studies [75,76,77], TARE may be considered an alternative treatment to TACE, particularly in larger HCCs, where the results are encouraging. However, this treatment modality requires further studies to confirm its real utility as a downstaging procedure [78]. The major limitation of downstaging protocols is that they can be applied only in a subgroup of patients who present simultaneously compensated cirrhosis complicated with HCC but cannot be applied in patients with decompensated cirrhosis with HCC.

A very challenging issue will soon be the potential role of neoadjuvant treatments combining systemic drugs such tyrosine kinase and checkpoint inhibitors in downstaging protocols [79]. In a recent clinical trial, the combination of atezolizumab plus bevacizumab in the treatment of unresectable HCC was able to induce a complete and partial response to therapy in 18% and 71% of treated patients, respectively [80]. These results open the critical question of whether systemic treatments may be adopted in combination and/or sequentially with traditional LRT to increase the chances of obtaining successful HCC downstaging.

A further critical element that must be considered in the application of downstaging procedures is the objective measurement of the treatment response. The modified Response Evaluation Criteria in Solid Tumors (mRECIST) were developed for the assessment of treatment response by measuring tumor shrinkage. These criteria divided the rate of response to treatment into four categories: (1) complete response (CR—disappearance of arterial enhancement in tumor(s), (2) partial response (PR—a minimum 30% reduction in the sum of diameters of viable tumors compared with baseline), (3) stable disease (SD—not meeting PR or progressive disease), and (4) progressive disease (PD—an increase of at least 20% in the sum of diameters of viable tumors compared with baseline or the appearance of new lesions) [81]. The usefulness of the mRECIST criteria in the evaluation of response to LRT after downstaging protocols in patients with HCC has been confirmed both in those within and beyond the MC. In a small series of 33 patients presenting HCC outside the MC who underwent LT after downstaging performed by TACE, the 5-year survival was significantly higher in those who achieved CR (94.4%) than in those who had PR (45.4%) and SD (50%). These significant differences were explained by a progressive increase in HCC recurrence rates from patients with CR (15.5%) to those with SD (50%) to those with PR (53.3%) [82]. Similar results were obtained in the study performed by Kim et al., which evaluated HCC recipients within and beyond MC after the TACE procedure [83]. The 5-year HCC recurrence rate was 5.3% in patients who achieved CR or PR after TACE compared with 17.6% in those who achieved SD or PD. It should be noted that although the mRECIST criteria are sufficiently detailed, they may not be systematically adopted among different transplant centers, such that the results obtained by LRT may not be comparable [19].

In addition to the quality of the response to LRT, the duration of response is increasingly used as a surrogate marker to identify HCC with more aggressive behavior. Starting from this assumption, many liver transplant centers adopted the strategy to “ablate and wait” to assess the type and duration of response to LRT [9]. It has been suggested that the success of downstaging should be assessed, demonstrating the absence of tumor progression during an observation period of at least 3 months after the procedure. A successful downstaging procedure allows the selection of candidates with more favorable tumor biology and better post-LT survival [84]. This strategy avoided early post-LT recurrences, as demonstrated in patients with HCC beyond MC transplanted after short waiting times [85]. Thus, the guidelines of both the American and European associations for the study of the liver are concordant in recommending the adoption of LRT in patients with HCC beyond MC and the consideration of those who achieved successful downstaging for at least 3–6 months as suitable candidates for LT [3,4,5,6,7,8,9,10,11,12,13,14,15,16,17,18,19,20,21,22,23,24,25,26,27,28,29,30,31,32,33,34,35,36,37,38,39,40,41,42,43,44,45,46,47,48,49,50,51,52,53,54,55,56,57,58,59,60,61,62,63,64,65,66,67,68,69,70,71,72,73,74,75,76,77,78,79,80,81,82,83,84,85,86].

The question at this time is what the baseline and the final burdens of HCC obtained after successful downstaging that may be considered sufficient to perform LT should be. In the United States, the UCSF downstaging protocol [70] has recently been employed as a national policy both for the graduation of urgency to transplantation and to try to answer this question. In this protocol, the baseline selection criteria for patients with HCC who may benefit from LRT downstaging procedures before LT were as follows: a single HCC ≤ 8 cm in diameter; up to three lesions < 5 cm in diameter; or up to five nodules, all of them <3 cm in diameter, but in any case with a total tumor diameter < 8 cm. Retrospectively analyzing the UNOS database, the 3819 liver transplanted patients with HCC were divided as always within the MC or achieved UNOS downstaging criteria (UNOS-DS). The 3-year post-LT survival was 83.2% in patients always within MC and 79.1% in those fulfilling UNOS-DS. Moreover, the 3-year HCC recurrence rate was 6.9% in recipients who were always within MC and 12.8% for those within UNOS-DS. A very interesting issue that emerged from this study was that AFP serum levels ≥ 100 ng/mL were the only independent predictor of post-LT HCC recurrence in downstaged groups [87].

To date, only one randomized clinical trial (the XXL trial) has evaluated the clinical outcome of patients presenting at baseline with HCC beyond MC who were successfully downstaged by means of LRT and subsequently transplanted compared with those who received only LRT [88]. This study was conducted in Italy and enrolled 74 patients with HCC beyond the MC, without macrovascular invasion or extrahepatic spread, with a 5-year expected post-LT survival of at least 50% (estimated by Metroticket calculator [37]) and preserved liver function (Child–Pugh classes A5–B7). All patients initially underwent tumor downstaging with LRT or systemic therapies, according to a multidisciplinary decision. After an observation period of 3 months, during which treatment with sorafenib was allowed, patients presenting CR or PR, assessed by means of the mRECIST criteria, were randomly assigned (1:1) to LT or to continue LRT or systemic treatments (control group). Of note, in patients with baseline AFP values ≥ 400 ng/mL, a radiological tumor response was confirmed only in case of a parallel percentage decrease in AFP concentrations. In contrast, in patients with AFP serum levels < 400 ng/mL at recruitment, an increase in AFP concentrations above that cutoff value at the end of the downstaging phase or during the observation period was considered tumor progression, independent of radiological assessment. The primary endpoints of the study were the evaluation of 5-year tumor-event-free survival and overall survival. The first result of the study was that 29/74 (39.1%) of the patients dropped out before randomization; thus, only 45 (60.9%) patients were finally included in the study (23 underwent LT, and 22 maintained LRT or systemic treatments). Despite the high rate of dropout, after a median follow-up of 71 months, a significantly higher 5-year tumor-event-free survival was observed in the LT group (76.8%) than in the control group (18.3%). Regarding the 5-year overall survival, the figures were quite similar, indicating a better overall survival in the LT group (77.5%) than in the control group (31.2%). Tumor progression was the main cause of death in both groups, while in the LT group, HCC recurrence was detected in 22% of cases. The results of this study provide additional evidence to previous results demonstrating comparable post-LT outcomes in patients with HCC beyond the MC who underwent successful downstaging within the MC and in those who underwent LT fulfilling the MC at presentation. A further very important message from this study is that different schedules of LRT and systemic treatments may be employed in patients with HCC beyond the MC to achieve successful and durable downstaging to permit them to be suitable for LT.

## 7. Conclusions

LT must be considered the best treatment option for patients with unresectable HCC [89]. Since the number of donors is insufficient to satisfy all requests for transplantation for HCC, it is essential to perform careful selection of transplant candidates. For approximately 25 years, MC have been the benchmark for offering patients with HCC the opportunity for transplantation, but as recently demonstrated, they excluded a subset of patients who could have benefited from LT. For this reason, several other more extended selection criteria to offer LT to an increasing number of patients with HCC have been evaluated. In the beginning, many of the expanded criteria evaluable in the pre-LT period were based on the morphological characteristics of the tumor as the original strategy adopted for constructing the MC. Subsequently, the addition of biological markers, predominantly AFP serum levels, to the morphological characteristics of the tumor emerged as the more solid and reproducible criteria for patient selection beyond the MC for LT, assuring excellent post-LT outcomes. Downstaging HCC to MC by means of LRT and/or systemic therapies is becoming a valid and increasingly utilized method of patient selection for LT. Adopting this approach, the surrogates of tumor biology can be assessed, such as the response rate to LRT and its maintenance for a sufficient time during the waiting list before transplant. The measurement of AFP serum levels or AFP slope during or after downstaging protocols can be considered a further important option to identify those patients at higher risk of HCC recurrence that should be excluded from LT. It appears clear that the risk of failure of successful downstaging is related to both the tumor burden and to AFP serum levels at baseline. 

It is important to highlight that the selection of HCC patients for LT by means of the expanded criteria may be difficult to adopt in areas of the world with severe organ shortages. In these areas, the selection criteria based on the utility principle that assures the maximum post-transplant survival, such as the MC, will remain preponderant, rather than expanded criteria that could reduce access to LT for patients with better post-LT prognosis [67,68,69,70,71,72,73,74,75,76,77,78,79,80,81,82,83,84,85,86,87,88,89,90]. This justified that not all liver transplant centers around the world adopt the same criteria to select patients with HCC for LT [3,4,5,6,7,8,9,10,11,12,13,14,15,16,17,18,19,20,21,22,23,24,25,26,27,28,29,30,31,32,33,34,35,36,37,38,39,40,41,42,43,44,45,46,47,48,49,50,51,52,53,54,55,56,57,58,59,60,61,62,63,64,65,66,67,68,69,70,71,72,73,74]. Each country developed, based on its scientific experiences, some selection criteria for transplanting patients with HCC beyond MC. A common thread that links the various selection criteria adopted in different countries is to consider the morphological characteristics of the tumor (number and size of nodules) and the values of some biological markers, mainly AFP, as main determinants of the selection process. In the United States and in Europe, the concept to not necessarily set a baseline HCC limit size to consider patients potentially transplantable, except for the presence of macrovascular invasion or extrahepatic spread, appears to be more accepted. Thus, the downstaging process will probably become the main selection tool for LT, enabling clinicians to postpone the transplantation decision from tumor presentation to the assessment of final response to LRT [91]. The effectiveness of downstaging procedures should be considered as having brought the tumor back within the MC for a period of at least 3–6 months before enlisting [84]. 

In summary, to try to answer the key questions reported in the introduction, the following proposals can be suggested: (a) it seems proven that in the absence of macrovascular invasion and extrahepatic spread, no upper limit of tumor burden beyond MC should be established “a priori” to determine transplant eligibility for HCC; (b) to justify a policy to transplant patients with HCC beyond MC at presentation, the minimal expected 5-year post-LT survival probability, estimated by Metroticket calculator, should be at least 50% [37]; (c) the response to neoadjuvant LRT and/or systemic treatments in addition to the dynamic evaluation of AFP serum levels are expected to replace conventional morphological criteria for selecting patients with HCC for LT in the near future.

## Figures and Tables

**Table 1:** The preoperative selection criteria for liver transplantation (LT) in patients with hepatocellular carcinoma (HCC) based on morphological characteristics of the tumor. Only the externally validated selection criteria are reported in the table.

Authors	Criterion Name	Country	No. of Patients	HCC Morphology	Post-LT Survival	Post-LT RFS
Mazzaferro et al. [8]	Milan	Italy	48	Up to 3 nodules <3 cm in diameter or up to 5 cm in diameter in the case of a single nodule	75% at 4 years	83% at 4 years
Fan et al. [20]	Shanghai Fudan	China	1078	Single nodule ≤ 9 cm in diameter, no more than 3 nodules with the largest ≤5 cm, a total tumor diameter ≤ 9 cm, without MVI or EHS	80% at 3 years	88% at 3 years
Yao et al. [14]	UCSF	US	168	Single nodule ≤ 6 cm in diameter or 2–3 nodules ≤ 4.5 cm, with a total tumor diameter ≤ 8 cm	-	80.7% at 5 years

RFS: recurrence-free survival; MVI: macrovascular invasion; EHS: extrahepatic spread; UCSF: University of California, San Francisco.

**Table 2:** The preoperative selection criteria for liver transplantation (LT) in patients with hepatocellular carcinoma (HCC) based on the addition of the biological serum markers and/or histological differentiation grade to the morphological characteristics of the tumor. Only the externally validated selection criteria are reported in the table.

Authors	Criterion Name	Country	No. of Patients	HCC Characteristics	Post-LT Survival	Post-LT RFS
Du Bay et al. [21]	Toronto	Canada	294	HCC confined to the liver, AFP serum levels <400 ng/mL, no poor histologic differentiation	70% at 5years	66% at 5 years
Zheng et al. [22]	Hangzhou	China	195	HCC ≤ 8 cm in diameter or >8 cm if associated with AFP serum levels < 400 ng/mL and histological grade I–II	70.7% at 5 years	62.4% at 5 years
Toso et al. [23]	Toso	Canada Swiss UK	233	Total tumor volume ≤ 115 cm^3^ and AFP serum levels ≤ 400 ng/mL	74.6% at 4 years	68% at 4 years
Duvoux et al. [24]	French	France	972	Nodule diameters ≤ 3 cm, between 3–6 cm, or ≥6 cm and AFP serum levels ≤ 100, between 100–1000, or >1000 ng/mL	69.9% at 5 years	66.6% at 5 years
Mazzaferro et al. [25]	Metroticket 2.0	Italy China	1359	The sum of the size (in cm) of the larger HCC and the number of nodules not exceeding 7, without MVI	74.9% at 5 years	77.9% at 5 years
* Kaido et al. [26]	Kyoto	Japan	198	Up to 10 HCCs with a diameter ≤ 5 cm and DCP serum levels ≤400 mAU/mL	82% at 5 years	-
* Lee et al. [27]	MoRAL	Korea	566	Simultaneous evaluation of DCP and AFP serum levels	86% at 5 years	66.3% at 5 years

RFS: recurrence-free survival; AFP: alpha-fetoprotein; MVI: macrovascular invasion; DCP: des-gamma-carboxyprothrombin. * The criteria were evaluated in living donor liver transplantation.
